# Acute Stroke at Term Pregnancy: What Should Happen Before the Epidural?

**DOI:** 10.7759/cureus.45613

**Published:** 2023-09-20

**Authors:** Sujeivan Mahendram, Kenneth Eichenbaum

**Affiliations:** 1 Anesthesiology, Wayne State University School of Medicine, Pontiac, USA; 2 Anesthesiology, Oakland University William Beaumont School of Medicine, Rochester, USA

**Keywords:** case report, quality improvement, patient safety, algorithm, acute stroke, labor analgesia

## Abstract

Acute stroke is a time-sensitive medical diagnosis, and current standardized management algorithms do not specifically streamline care for pregnant patients with these symptoms. Here, we discuss the management of a 29-year-old parturient with a history of systemic lupus erythematosus (SLE) who presented with stroke-like symptoms. We discuss strategies to improve care by incorporating formal neurological and ophthalmologic evaluations prior to referral for neuraxial intervention, particularly in light of the developing concerns among ophthalmologists that retinal transient ischemic attacks (TIAs) and visual symptoms should be treated with the same acuity as cerebral TIAs and strokes. We propose an integrated stroke algorithm in the pregnant population with consideration for specific ophthalmologic evaluation. In the present case, labor induction and epidural placement were successfully performed once a more optimized workup was completed.

## Introduction

The incidence of stroke in young and middle-aged individuals is increasing and should be regarded as more than just a comorbidity of the elderly population [1]. A recent systematic review and meta-analysis reported the incidence of stroke to be as high as 30 per 100,000 pregnancies, which is approximately three times that seen in nonpregnant females [2]. With this in mind, it is important to have a framework in place that allows a prompt workup in conjunction with appropriate consultations in order to safely manage both the mother and baby. Currently, most hospital protocols for acute stroke incorporate a standardized algorithm for the management of acute stroke in the adult population. However, there is a paucity of literature that maps out a step-by-step plan for parturients presenting with stroke-like symptoms. Written Health Insurance Portability and Accountability Act of 1996 (HIPAA) authorization for the publication of this case report was obtained from the patient discussed herein.

## Case presentation

We present a case of a 29-year-old gravida 2, para 1 (G2P1) at 38.5 weeks of gestational age who was admitted for sudden-onset visual changes associated with slurred speech and right-sided arm weakness. Her symptoms were present for approximately 25 minutes prior to arrival. The patient was brought to the emergency department (ED) where a CT of the head identified no obvious signs of acute stroke. However, while the radiology report recommended further imaging and neurological workup, the ED transferred the patient for prompt evaluation by both obstetrics (OB) and maternal-fetal medicine (MFM) services. The patient was admitted to labor and delivery for overnight observation. MFM recommended the induction of labor, stating that expedited delivery would be optimal for the safety of both the mother and baby. The augmentation of labor was initiated by the obstetrics team, and the anesthesia team was asked to place a labor epidural.

During the pre-anesthetic assessment, the patient endorsed a recurrence of visual disturbance and complained of episodic blurry vision since admission. Given that no neurology consult had been performed and with continued and evolving symptoms, the anesthesia team requested the discontinuance of the labor induction pending neurological evaluation. This request was discussed with the patient and spouse along with the OB/MFM teams. There was general agreement that neurology consultation was necessary and beneficial prior to proceeding with further induction of labor. With distinct visual symptoms, the option of a formal ophthalmologic evaluation was considered but not implemented. With ongoing symptoms and the need for emergent evaluation, a stroke code was called for immediate neurological assessment. The neurologist endorsed further imaging with magnetic resonance imaging (MRI) of the brain prior to reinitiating labor induction. MRI was unremarkable for acute infarct or stroke, and there was no significant mass effect or intracranial obstruction noted. Neurology recommended no further neurological intervention given the interval resolution of the patient’s symptoms. The patient subsequently received a labor epidural and completed an uncomplicated spontaneous vaginal delivery.

## Discussion

Acute-onset slurring of speech and visual compromise cause immediate concern for an acute cerebrovascular accident. In the setting of new stroke-like symptoms combined with the procoagulant state of pregnancy, a prompt in-person neurological evaluation is critical. These symptoms in parturients may be more prevalent given the coagulopathic physiology associated with pregnancy. Of note, in the case of our patient, there was a known medical history of migraine with aura and systemic lupus erythematosus (SLE), which can complicate the etiology of her presentation. Thromboembolic disease is a known complication of SLE [3-5]. Giorgi et al. reviewed a variety of transient visual changes associated with SLE [6]. In addition, there is an increased risk of permanent blindness [7]. With this in mind, there are new recommendations suggesting that an emergent ophthalmologic consult should be performed [8].

**Figure 1:**
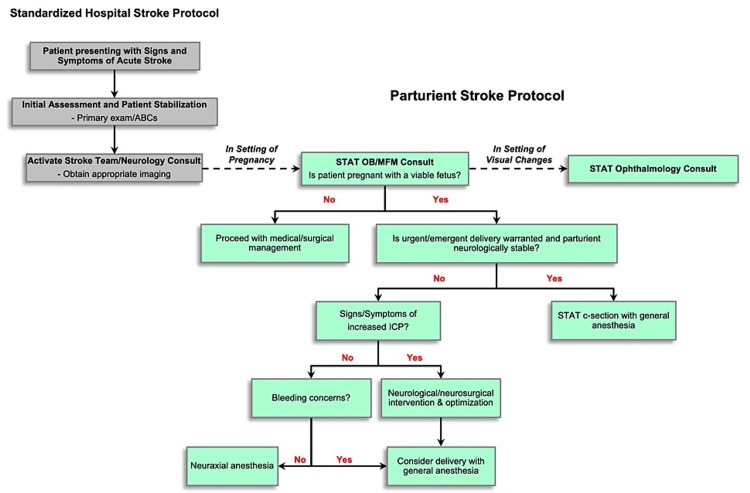
Proposed algorithm for the management of suspected adult stroke in the parturient. ABC, airway, breathing, and circulation; OB, obstetrics; MFM, maternal-fetal medicine; ICP, intracranial pressure

After a thorough workup, the neurologist felt that the likelihood of embolic stroke in this patient was unlikely, and this patient was diagnosed with a reversible complex migraine manifesting as a transient ischemic attack (TIA) without evolution into an ischemic infarct. A more comprehensive workup in a non-emergent setting may include further studies such as an echocardiogram and/or transcranial Doppler with bubble study. There is no conclusive data in the literature that suggests a superior mode of delivery (i.e., vaginal versus cesarean section) following acute stroke. However, several case studies recommend early epidural placement in laboring parturients to facilitate normotension and to minimize Valsalva, thereby preventing increased intracranial pressure [9,10]. Ultimately, a multidisciplinary and patient-specific approach should be incorporated into patient care when determining the mode of delivery. For this reason, we propose that an updated algorithm (Figure 1) be incorporated into current acute stroke protocols [11]. The proposed algorithm not only emphasizes a methodical anesthetic approach to managing parturients who present with stroke-like symptoms but also necessitates an ophthalmology consultation in the setting of acute visual changes. This may help avoid patient handoffs with suboptimal management and without obligate expert evaluation.

## Conclusions

Overall, caring for a parturient in the setting of acute stroke poses the challenge of treating two patients whose medical courses are closely linked. It is necessary to carry out a rapid and comprehensive workup of such patients being admitted via the ED, along with a multidisciplinary discussion with OB, MFM, neurology, and ophthalmology teams. Fortunately for our patient, the symptoms resolved postdelivery with no further complications.
